# An Analysis Model for Studying the Determinants of Throwing Scoring Actions During Standing Judo

**DOI:** 10.3390/sports7020042

**Published:** 2019-02-15

**Authors:** Xian Mayo, Xurxo Dopico-Calvo, Eliseo Iglesias-Soler

**Affiliations:** 1 Observatory of Healthy and Active Living of Spain Active Foundation, Centre for Sport Studies, King Juan Carlos University, 28942 Madrid, Spain; 2 Performance and Health Group, Department of Physical Education and Sport, Faculty of Sports Sciences and Physical Education, University of A Coruna, 15179 A Coruña, Spain; xurxo.dopico@udc.es (X.D.-C.); eliseo@udc.es (E.I.-S.)

**Keywords:** combat strategy, technical analysis, scoring performance, notational analysis

## Abstract

In judo, the attacking system is grounded on several determinants of the chances to throw. In our study, data regarding four determinants of the attacking system were collected in order to classify the standing scoring actions: the attacking type (direct/counter-attack), the throwing area (forward/backward), the technique’s category (based on motor criteria), and the lateral structure of fighting (contenders with a symmetrical/asymmetrical position). To study the usefulness of such an analysis, the standing scoring actions of the 2013 Judo World Championship were analyzed as an example of elite judo’s attacking system (*n* = 775). The Pearson’s chi-squared test and Cramér’s V were used to analyze the hypothesis of a uniform distribution or the association between variables and the strength of such an association, respectively. The scoring actions (*p* < 0.001) were mostly direct attacks (82.6%), in the forward throw area (57.5%), and in an asymmetrical position (67.2%). All of the variables were associated (*p* < 0.05; V = 0.11–0.54), with higher proportions of counter-attacks and attacks occurring on the backward thrown area during asymmetrical structures than the expected. Some categories of techniques were observed more than expected, depending on the symmetrical or asymmetrical structure. Our data augment the knowledge of standing judo by showing features of the attacking system, suggesting strategies for optimizing performance.

## 1. Introduction

Success in judo depends on some variables that are technical- and tactical-related and that determine the unpredictability [1,2,3] and effectiveness [4,5,6] during combat. In this sense, important variables reported in the literature while performing standing judo (i.e., nage waza) that may determine success in a combat are related with gripping—such as gripping variability, gripping contest time, and gripping contact time [7], versatility and technical variation [6,8], and throwing directions of such techniques [8,9,10]. The interrelatedness of building of these variables altogether in a methodical way and being theoretically integrated is what is called an attacking system [2,9]. The attacking system can be resumed in two different kinds of strategies that work together during combat to maximize the chances of scoring and thus winning. Firstly, the judoka can try to perform different techniques from the same grip. Secondly, the judoka can try to perform the same technique from different grips [8].

In this sense, after the gripping contest, and once that the opponent is gripped, a judo athlete starts an action or a series of actions that will be defined by the attacking system. This attacking system will be structured on several a priori or evolving decisions throughout the combat that would increase the chances of scoring and win. Among others, we can find (a) the grade of offensiveness, (b) the throwing area, (c) the versatility of techniques used, and (d) the lateral structure of fighting of the judo athlete in relation to the opponent.

Regarding these variables, the grade of offensiveness stands for the tactical decision in which the judo athlete would decide to try direct scoring or focusing more on counter-attacks [11]. The throwing area is defined by the space in which the judo athlete is trying to throw the opponent, which is determined by the selected techniques and the particular contextual factors of the combat. In this regard, it has been previously shown that increasing the possible throwing directions augments the chances of scoring [8,9]. The versatility of the judo athlete is defined by the techniques that are preferably performed (i.e., tokui waza) and the associated throwings with those particular techniques that are based on its proficiency and creativity [6]. The lateral structure of fighting refers to the lateral stance of contenders. Thus, the lateral structure can be symmetric if the lateral stance is the same for both contenders (e.g., right stance versus right stance) and/or asymmetric if the lateral stance is different (e.g., right stance versus left stance).

While there are other factors that may improve the chances of scoring and winning, such as improving groundwork judo (i.e., osae, shime or kansetsu waza) or focusing on achieving penalties on the adversary (i.e., shido), the core attacking system is more likely to be defined around those four aforementioned variables. Nevertheless, previous observational studies failed to report the reproducible analyses of some variables, while explaining the attacking system. This was true for both the throwing area while considering all the possible directions or areas of throwing [12,13] and for the categorization of the techniques when it was performed following the Kodokan structure [14], given its limitations for training purposes.

In this sense, analyses with more robust levels of agreement are needed to be able to close the gap between research and coaching delivery. In this regard, previous studies analyzing the techniques being categorized on motor criteria [15] or the lateral structure of fighting based on symmetry and asymmetry [4] have been carried out showing strong intra-observer levels of agreement (κ > 0.9). Thus, observational analyses should preferentially use these sorts of variables when analyzing judo combats in order to obtain more reliable data. Thereby, observational analysis following motor criteria will be more comprehensive than anatomical (i.e., the Kodokan structure) [16] or mechanical [17] criteria, allowing for a more robust approach from a motor control viewpoint and a better explanation of the tactical requirements from a spatial and cognitive standpoint.

In this regard, a previous study analyzing combats that was based on the lateral preference found out that symmetry lateral preference between contenders provided a higher likelihood of winning in comparison with an asymmetrical lateral preference [4]. Nevertheless, this would be contrary to the “fighting hypothesis”, which stands the strategic advantages of left-handiness against a right-hander during a combat due to unfamiliarity, since throws would occur from unexpected directions and angles [18]. It is important to note that this previous report merged the actions of both left- and right-handers in a general asymmetrical lateral preference group without subgroups, thus probably including noise in the analysis [4].

The aim of this study was to analyze the usefulness of four determinants in standing judo, such as the attacking type (i.e., direct or counter-attack scoring), the throwing area (forward or backward throw area), the category of scoring actions (based on motor criteria), and the lateral structure of fighting for analyzing the attacking system that is performed in judo competitions. To do that, an example analysis was carried with data that were obtained by video observation of the 2013 Judo World Championship throwing scoring actions.

Our hypothesis is that, will be “sweet spots”, which will increase the chances of scoring when considering the association between the four variables explained. Besides, there will be some group of techniques that will better work during symmetrical or asymmetrical positions, therefore being position-dependent. The data arising from the performance analysis of scoring of this study will help coaches and judo athletes to plan combat strategies and design suitable training situations. In this sense, it is important to note that strategies and training drills should be designed when considering real competition scoring to being able to be both effective and efficient. As a consequence of this, the kind of analysis that is focused on tactical scoring pattern would be better suited than frequency-based analysis, which is just focused on techniques that are performed during combat [3].

## 2. Materials and Methods

### 2.1. Experimental Design

In order to analyze the influence of some determinants of the attacking system on the scoring actions during standing judo, the 2013 Judo World Championship data were considered. During the 2013 Judo World Championship, which was celebrated in Rio de Janeiro, all individual combats for both women and men were investigated by video analysis and the scoring actions were obtained. The championship analyzed involved a total of 14 tournaments, structured by gender and weight (women: −48 kg, −52 kg, −57 kg, −63 kg, −70 kg, −78 kg, and +78 kg; men: −60 kg, −66 kg, −73 kg, −81 kg, −90 kg, −100 kg, and +100 kg), in which a total of 680 athletes were analyzed (260 women and 420 men). From all the tournaments and combats, a total of 755 scoring actions were collected, in which 279 and 476 were performed by women and men, respectively.

### 2.2. Structure of Scoring Actions

Only actions that were scored during standing judo (i.e., nage waza) were considered. Briefly, previously to 2017, there were three possible scores during a combat—ippon, waza-ari, and yuko. When the first ippon was scored, the combat was finished. Two waza-ari were equivalent to an ippon, and thus the combat was also finished. Lastly, there were no limits in scoring as many yuko as possible. The athlete who scored an ippon or two waza-ari won and if an ippon or two waza-ari were not scored, then the athlete with the higher punctuation (i.e., waza-ari, then yuko) won. The scoring actions collected were structured in attacking type, throwing area, the category of scoring actions, and the lateral structure of fighting.

#### 2.2.1. Attacking Type of the Scoring Action

Scoring actions regarding the attacking type were structured in direct scoring, when the score is the effect of a technique that was attempted by its own or counter-attack scoring, when the score is the effect of a technique that is the reaction of a movement capitalized from an opponent [11].

#### 2.2.2. Throwing Area of the Scoring Action

Scoring actions regarding the throwing area were structured in forward throw area or backward throw area [13].

#### 2.2.3. Category of the Techniques

The categorization of scoring actions was carried out based on the motor criteria that were published elsewhere [16]. Briefly, these criteria organized the techniques when considering the common motor features, which are indispensable in creating an adaptive motor program [16]. Thus, techniques can be organized based (a) on the movement structure (i.e., techniques with turning before the execution, techniques without turning before the execution, or techniques that are performed during supine position), (b) on the direction of the dynamic leg (i.e., ipsilateral direction or contralateral direction), (c) on the spatial zone of the dynamic leg (i.e., inner zone or external zone), (d) on the space where the opponent is thrown (i.e., forward throw area or backward throw area), and (e) on the sustentation base (i.e., one support, two supports, or back support).

This categorization gives nine groups of techniques that are based on the aforementioned motor criteria, which are: (1) Techniques of turning, forward throw, and two supporting legs (Turn_F2; e.g., o goshi, uki goshi, etc.); (2) Techniques of turning, forward throw, and one supporting leg (Turn_F1; e.g., uchi mata, harai goshi, etc.); (3) Techniques without turning, ipsilateral leg direction, external zone, backward throw, and one supporting leg (WT_IpExB1; e.g., o soto gari, o soto gake, etc.); (4) Techniques without turning, ipsilateral leg direction, inner zone, backward throw, and one supporting leg (WT_IpInB1; e.g., ko uchi gari, ko uchi gake, etc.); (5) Techniques without turning, contralateral leg direction, inner zone, backward throw, and one supporting leg (WT_ClaInB1; e.g., o uchi gari, o uchi gake, etc.); (6) Techniques without turning, contralateral leg direction, external zone, backward throw, and one supporting leg (WT_ClaExB1; e.g., ko soto gake, de ashi harai, etc.); (7) Techniques without turning, contralateral leg direction, external zone, forward throw, and one supporting leg (WT_ClaExF1; e.g., sasae tsuri komi ashi, hiza guruma, etc.); (8) Techniques without turning, forward or backward throw, and two supporting legs (WT_2; e.g., ura nage, ushiro goshi, etc.); (9) Techniques without turning, forward or backward throw, and one supporting leg (WT_1; e.g., uchi mata sukashi, etc.); and, (10) Techniques of supine position, forward throw, and back support (SP_FwBack; e.g., tomoe nage, sumi gaeshi, etc.). A clear overview of these techniques’ categorization with the defined list of techniques following the Kodokan traditional judo techniques organization can be observed in Dopico et al. (2014, p. 81, Table 1) [16].

#### 2.2.4. Lateral Structure of Fighting During a Scoring Action

In a judo athlete and during a scoring action, there are two possible lateral structures of fighting (i.e., stances) that are adaptive throughout combat [8,19], which are in disregard of the primary posture (i.e., general posture during combat) [20]. Thus, and for the sake of this study, a judo athlete can take a right lateral structure of fighting (R) or a left lateral structure of fighting (L) when is executing or receiving a scoring action [12]. While executing a scoring action, these two possibilities are defined by the movement structure, i.e., (a) during a technique with turn before execution, the scoring action is considered to be performed such as with an R when the rotation of the right shoulder turns to the left (anticlockwise), and such as with an L when the rotation of the left shoulder turns to the right (clockwise); (b) during a scoring technique without any turn before execution, the scoring action is considered such as with an R if the dynamic leg (i.e., the leg that is reaping) is the right one, and such as with an L if the dynamic leg is the left one [16]. While receiving a scoring action, the stance is considered such as with an R when the one who is receiving the scoring action (i.e., uke) has the right foot is advanced and such as with an L when uke has its right foot is advanced.

Therefore, the scoring actions that are based on the lateral structure of fighting are defined for this study such as symmetrical, if both competitors executed and received a scoring technique with an R or L; or asymmetrical if the competitors executed and received a scoring technique in different stance sides.

Accordingly, and on the one hand, symmetrical positions would have an R-R or L-L structure, in which the first letter represents the lateral structure of fighting of the one performing the scoring action (i.e., tori) and the second letter represents the stance of the one that is receiving the scoring action (i.e., uke). On the other hand, asymmetrical positions would have an R-L or L-R structure, depending in which tori is performing the scoring action, such as right- or left-handed, respectively, and uke has a left- or right-handed stance receiving the scoring action, respectively.

### 2.3. Statistical Analysis

The public TV broadcast of the 2013 Judo World Championship was analyzed with the program Lince 1.4 and all of the scoring actions were collected with an ad-hoc instrument that was created specifically for this analysis. These scoring actions were structured in four determinants of standing judo, i.e., attacking type (direct scoring and counter-attack scoring), the throwing space (forward throw area and backward thrown area), the category of the technique (Turn_F2, Turn_F1, WT_IpExB1, WT_IpInB1, WT_ClaInB1, WT_ClaExB1, WT_ClaExF1, WT_2, WT_1, and SP_FwBack), and the lateral structure of fighting (symmetrical [R-R and L-L] and asymmetrical [R-L and L-R]) of such scoring actions. Two researchers (X.D. and I.S.) with the Kappa coefficient (κ = 0.92) carried out a concordance analysis to analyze the agreement between the measures. To analyze the scoring actions, a Pearson’s chi-squared (χ2) of one sample test was implemented in order to analyze the hypothesis of a uniform distribution of dichotomic variables (50%). Pearson’s chi-squared (χ2) was used in order to analyze the association between the four variables. For analyzing the strength of the association, Cramér’s V was implemented. Additionally, the odds ratio (OR) with 95% of confidence intervals (CI) were calculated for analyzing the effect size of meaningful comparisons in 2 × 2 contingency tables. The level of significance was set at 0.05 and the data are presented as percentages (%).

## 3. Results

As can be observed in Table 1 and Table 2, there was a higher proportion of scoring actions being direct, χ² (1) = 321.92, occurring in the forward throw area, χ² (1) = 17.24, and in an asymmetrical structure of fighting, χ² (1) = 88.85, in comparison with the proportion of scoring actions of counter-attacks, occurring in the backward throw area, and in a symmetrical structure of fighting (*p* < 0.001).

### 3.1. Attacking Type by the Throwing Space

As can be observed in Table 1, the relation between the attacking type and the throwing space was significant, χ² (2) = 77.49, Cramér’s V = 0.32; *p* < 0.001. In this regard, the standardized residuals showed that the observed proportion of counter-attacks that occurred in the backward throw area was higher than the expected proportion, while the observed proportion of counter-attacks that occurred in the forward throw area were lower than the expected proportion. In this regard, the odds for a counter-attack occurring in a backward thrown area were higher than the odds for a counter-attack occurring in a forward throw area (OR 5.8, 95% CI: 3.8–9.0).

### 3.2. Attacking Type by the Lateral Structure of Fighting

There was an association between the attacking type and the lateral structure of fighting (symmetrical or asymmetrical), χ² (1) = 30.59, Cramér’s V = 0.20; *p* < 0.001. In this regard, and as can be observed in Table 2, the standardized residuals showed that the observed proportions of counter-attacks occurring in the asymmetrical lateral structure of fighting were more than the expected proportion, while the observed proportion of counter-attacks occurring in the symmetric position were less than the expected proportion. Thus, the odds for a counter-attack scoring action occurring in an asymmetrical position were higher than a counter-attack scoring action occurring in a symmetrical position (OR 4.3, 95% CI: 2.5–7.4).

When analyzing the subgroups and as illustrated in Table 2, there was also an association between the attacking type and the lateral structure of fighting subgroups (R-R, L-L, R-L, and L-R), χ² (3) = 36.61, Cramér’s V = 0.22; *p* < 0.001. In this regard, the standardized residuals showed that the observed proportion of counter-attacks that occurred in the R-L lateral structure of fighting were higher than the expected proportion, while the observed proportions of the counter-attacks that occurred in the R-R lateral structure of fighting were lower than the expected proportion. In this regard, the odds for a counter-attack scoring action occurring in an R-L position were higher than a counter-attack scoring action occurring in an R-R position (OR 8.6, 95% CI: 3.6–20.5).

### 3.3. Lateral Structure of Fighting by the Throwing Space

There was an association between the lateral structure of fighting (symmetrical or asymmetrical) and the throwing space, χ² (2) = 8.2, Cramér’s V = 0.1; *p* = 0.04. Nevertheless, and as reflected in Table 3, the standardized residuals did not show any observed proportion higher or lower than the expected. Nonetheless, the odds for a scoring action occurring in the backward area and in an asymmetrical position were higher than a scoring action occurring in a symmetrical position (OR 1.6, 95% CI: 1.2–4.0).

When analyzing the subgroups and as Table 3 points out, there was also an association between the lateral structure of fighting subgroups (R-R, L-L, R-L, and L-R) and the throwing space, χ² (3) = 9.56, Cramér’s V = 0.11; *p* = 0.023. Nevertheless, the standardized residuals did not indicate that any observed proportion as higher or lower than the expected. When comparing the subgroups within the symmetrical and the asymmetrical comparisons and with R executors, the odds for scoring throws in the backward area occurring with an R-L lateral structure of fighting were higher than the scoring throws occurring with an R-R lateral structure of fighting (OR 1.8, 95% CI: 1.2–2.8).

### 3.4. Lateral Structure of Fighting by the Category of Techniques

There was an association between the lateral structure of fighting (symmetrical or asymmetrical) and the category of techniques, χ² (9) = 227.5, Cramér’s V = 0.54; *p* < 0.001. In this regard, and as can be observed in Table 4, the standardized residuals showed that the observed proportions with the symmetrical lateral structure of fighting, when considering the categories of techniques, were higher than expected for Turn_F2, WT_ClaExF1, WT_IpExB1, and WT_IpInB1 and lower for Turn_F1, WT_2, WT_ClaExB1, and WT_ClaInB1. Additionally, the standardized residuals showed that the observed proportions with the asymmetrical lateral structure of fighting, when considering the categories of techniques, were higher than expected for WT_2, WT_ClaExB1, and WT_ClaInB1 and lower than expected for Turn_F2, WT_ClaExF1, WT_IpExB1, and WT_IpInB1.

When analyzing the subgroups and as denoted in Table 4, there was also an association between the lateral structure of fighting subgroups (R-R, L-L, R-L, and L-R) and the category of techniques, χ² (27) = 246.70, Cramér’s V = 0.57; *p* < 0.001. In this regard and regarding the symmetrical subgroups, the residuals showed that the observed proportions with the R-R lateral structure of fighting, when considering the categories of techniques. were higher than the expected for Turn_F2, WT_ClaExF1, and WT_IpExB1 and lower than the expected for WT_2, WT_ClaExB1, and WT_ClaInB1. With the L-L lateral structure of fighting, higher proportions than the expected were observed for WT_ClaExF1, WT_IpExB1, and WT_IpInB1 and lower proportions than the expected were noted for Turn_1, WT_2, WT_ClaExB1, and WT_ClaInB1.

**Figure 1:**
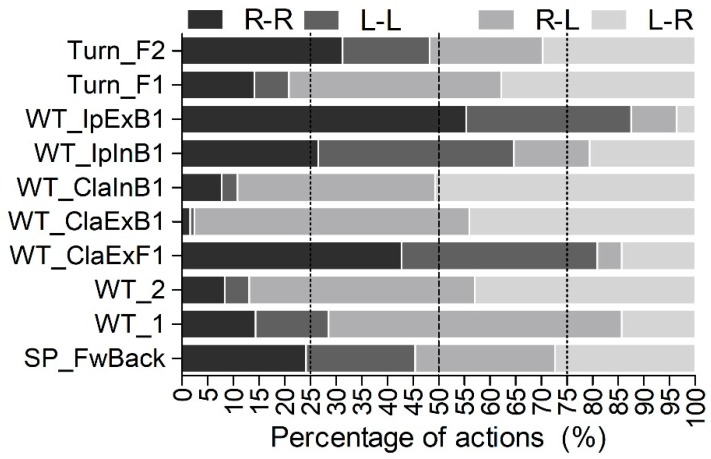
Graphical representation of the percentages of scoring actions of the different lateral structure of fighting (in dark grey, symmetrical structures [R-R and L-L] and in light gray, asymmetrical structures [R-L and L-R]) regarding the category of technique (*n* = 755). R and L represent a right lateral structure of fighting or a left lateral structure of fighting, respectively. The first letter represents the lateral structure of fighting of the judo athlete performing the action while the second letter represents the lateral structure of fighting of the judo athlete receiving the action. Turn_F2: Techniques of turning, forward throw, and two supporting legs; Turn_F1: Techniques of turning, forward throw, and one supporting leg; WT_IpExB1: Techniques without turning, ipsilateral leg direction, external zone, backward throw, and one supporting leg; WT_IpInB1: Techniques without turning, ipsilateral leg direction, inner zone, backward throw, and one supporting leg. WT_ClaInB1: Techniques without turning, contralateral leg direction, inner zone, backward throw, and one supporting leg; WT_ClaExB1: Techniques without turning, contralateral leg direction, external zone, backward throw, and one supporting leg; WT_ClaExF1: Techniques without turning, contralateral leg direction, external zone, forward throw, and one supporting leg; WT_2: Techniques without turning, forward or backward throw, and two supporting legs; WT_1: Techniques without turning, forward or backward throw, and one supporting leg; SP_FwBack: Techniques of supine position, forward throw, and back support.

Regarding the asymmetrical subgroups, with the R-L lateral structure of fighting, higher proportions than expected were observed for WT_ClaExB1 and lower proportions than the expected were reported for Turn_F2, WT_ClaExF1, and WT_IpExB1. Lastly, with the L-R lateral structure of fighting, higher proportions than the expected were observed for WT_ClaExB1 and WT_ClaInB1 and lower proportions than the expected were noted for WT_IpExB1. A graphical representation of the percentages of scoring actions in the different lateral structure of fighting regarding the category of technique can be observed in Figure 1.

## 4. Discussion

Our data point out that most of the scoring actions in standing judo in the data analyzed were direct attacks, occurring in the forward throw area, and in an asymmetrical lateral structure of fighting. In this regard, there were a higher proportion of counter-attacks that occurred in the backward throw area and in an asymmetrical lateral structure of fighting than expected. When considering the lateral structure of fighting and the category of technique, during the symmetrical lateral structures of fighting, there was a higher proportion of scoring actions than the expected for the categories Turn_F2, WT_ClaExF1, WT_IpExB1, and WT_IpInB1. On the other hand, during the asymmetrical lateral structures of fighting, there was a higher proportion of scoring actions than expected for the categories WT_2, WT_ClaExB1, and WT_ClaInB1. When considering the subgroups, higher proportions were observed for Turn_F2, WT_ClaExF1, and WT_IpExB1 during R-R, WT_ClaExF1, WT_IpExB1, and WT_IpInB1 during L-L, WT_ClaExB1 during R-L, and WT_ClaExB1 and WT_ClaInB1 during L-R.

Our results agree with a previous study analyzing the attacking type in senior Japanese judo athletes during the 2010 World Judo Championship, in which the authors found a greater percentage of direct attack actions (66.6%) in comparison with the counter-attacks (33.3%) [11]. When these data are less extreme than ours (82.6% of direct attacks versus 17.4% of counter-attacks), it is important to point out that, while they analyzed effective and ineffective attacks together, we just analyzed the scoring attacks.

In our analysis, a higher proportion of scoring actions occurred in the forward throw area in comparison with the backward throw area, which is also coincident with the literature [12,21]. On the one hand, it was observed that, during the 2010 World Judo Championship, both Japanese judo men athletes and their opponents performed a higher number of throws in the forward throw area in comparison with the backward throw area [21]. On the other hand, during an analysis of 12 international judo athletes, ten of them were more effective scoring in the forward throw area than in the backward thrown area [12]. In this regard, it is important to note that, in the literature, there are diverse ways of organizing the throwing space. For example, the throwing space can be organized in a forward and backward throw area, such as in our study and in another one that was previously analyzed [21]. In this sense, we used a dichotomic structure to look out for higher levels of replicability and because a simpler organization allows for a stronger cohesion from a motor control standpoint and better explains the tactical needs from a spatial and cognitive point of view [16]. In this sense, previous attempts to analyze this variable showed low levels of replicability in some directions when using four possible attacking areas (back, front, right, and left) [3] or eight possible throwing areas (back, back right, back left, front, front right, front left, right, and left) [13].

Respecting the lateral structure of fighting, the asymmetrical lateral structure of fighting accounted for the 67.1% of the scoring actions, in which both R-L and L-R virtually represented the same scoring actions. This is consistent with a previous article that found that judo athletes have a significant correlation between the efficiency during the combat as R and L executors against L and R receivers, respectively (i.e., R-L and L-R), but not during R-R and L-L combats [12]. This would point out the ease of scoring in an asymmetrical lateral structure of fighting in comparison to a situation of a symmetrical lateral structure of fighting. However, contrary to our data and to the “fighting hypothesis”, another previous study analyzing gripping reported that a symmetrical lateral preference gives higher attack effectiveness and a higher likelihood of winning in comparison with an asymmetrical lateral preference [4]. Nevertheless, in disregard of the scoring actions, they reported that the asymmetrical lateral structure of fighting was more frequent during the combats [4]. Two differences arise between our study and theirs: Firstly, they did not analyze the scoring actions, but rather the total actions, the effectiveness of actions, and the chances of winning. Secondly, they did not analyze the lateral preference of fighting during the throws per se, but rather the lateral preference of the gripping, thus being possible to match or not match the relation grip-throw while scoring.

Regarding the lateral structure of fighting by the throwing space, it is important to note that, during the asymmetrical lateral structure of fighting, we observed a higher proportion of actions occurring in the backward throw area, while also observing a lower proportion of actions occurring in the forward throw area in comparison with the expected. Interestingly, in the article of Adam et al. [4], they found a significant correlation during the asymmetrical lateral preference of fighting on the efficiency between the left backward throw area and the left forward throw area of the opponent (L receiver) during combats.

Regarding the category of scoring actions, a previous time-based attempt to perform an analysis that was based on the Kodokan structure reported low reproducibility levels [14]. Additionally, the studies in the literature reporting on the frequency of techniques used while attacking or scoring are scarce [17,21]. When considering both the 2010 World Judo Championship Japanese judo men athletes [21] and the 2012 Summer Olympic Judo Championship [17], and the techniques are structured based on the motor criteria, the categories reported and their percentages broadly match with our data.

Interesting data arise from our study, such that some categories of scoring actions are particularly effective for different lateral structures of fighting, in our analysis showing higher proportions than expected. This was the case during symmetric situations for WT_2, WT_ClaExB1, and WT_ClaInB1. During asymmetric situations, some groups were extremely useful, such as Turn_F2, WT_ClaExF1, WT_IpExB1, and WT_IpInB1. Additionally, it seems to be “sweet spots” for particular situations of the lateral structures’ subgroups, such as the case of Turn_F2, WT_ClaExF1, and WT_IpExB1 during R-R, WT_ClaExF1, WT_IpExB1, and WT_IpInB1 during L-L, WT_ClaExB1 during R-L, and WT_ClaExB1 and WT_ClaInB1 during L-R. This range of possibilities during a confrontation has a stronger practical utility and it should be implemented in an individual approach to technical-tactical preparation by the coaches to increase the chances of scoring and thus winning. The better understanding of standing judo that arises from this study when analyzing the scoring actions instead of the frequency of actions will consider the real competition needs better and thus help to create better training drills. This, at the same time, will help to define better-attacking systems by creating more proficient combat strategies.

There are two potential limitations that should be noted. Firstly, in our study, we collected together the scoring actions of men and women of different weight categories to analyze such actions. It is possible that some differences exist between genders [17,22] and weight categories [17], since some discrepancies were previously reported. In this sense, we agree with a previous report pointing out that every category may have specific characteristics and preferred methods of attack [23]. Secondly, some changes in refereeing occurred from 2013 to the present that lightly may alter our results. Nevertheless, we think that neither merging the data nor the changes in refereeing have a significant effect on the outcomes of our study or on its applicability to competition.

## 5. Conclusions

In summary, our results showed that the determinants of the attacking system that were considered in this study allowed for a solid analysis of standing scoring actions in judo. In these data, there was a preponderance of scoring actions that were direct attack scores, occurring in the forward throw area, and in an asymmetrical lateral structure of fighting. Additionally, there were a higher proportion of counter-attacks and attacks that occurred on the backward thrown area during the asymmetrical lateral structures of fighting in comparison with the symmetrical lateral structures of fighting.

Subsequently, some groups were particularly effective during concrete structures of fighting. In this sense, a higher proportion of scoring actions of the categories Turn_F2, WT_ClaExF1, WT_IpExB1, and WT_IpInB1 were reported during the symmetrical lateral structures of fighting, while a higher preponderance of actions of the categories WT_2, WT_ClaExB1, and WT_ClaInB1 were collected during the asymmetrical lateral structure of fighting when compared with the expected. While considering subgroups, some categories are definitively more effective due to particular preponderances of observed categories versus the expected, such are the cases of Turn_F2, WT_ClaExF1, and WT_IpExB1 during R-R, WT_ClaExF1, WT_IpExB1, and WT_IpInB1 during L-L, WT_ClaExB1 during R-L, and WT_ClaExB1 and WT_ClaInB1 during L-R.

The outcomes of this study will provide a deeper knowledge regarding the scoring actions that occur in standing judo. In this sense, it will let coaches deliver combat strategies and training situations, helping judo athletes to take advantage of specific setting during the combat. As a consequence of this, they will be able to exploit specific situations when considering the attacking type, the throwing area, the lateral structure of fighting, and thus the range of possible categories of techniques. Additionally, further analyses using this methodology should analyze more recent data in order to ascertain the tenability of the findings that were obtained with these data. Implementing these outcomes in real training will afterwards increase the chances of scoring and winning by the judo athletes.

## Figures and Tables

**Table 1:** Relation between the attacking type (direct actions or counter-attacks) and the throwing space (forward or backward throw area) (*n* = 754).

			Attacking Type		
			Direct Actions	Counter-Attacks	Total
Throwing space	Backward throw area	Number of actions	221	99	320
		Percentage within throwing space	69.1%	30.9%	
		Percentage within attacking type	35.4%	75.6%	42.4%
		Total percentage	29.3%	13.1%	
		Standardized residuals	−2.7	5.8	
	Forward throw area	Number of actions	403	31	434
		Percentage within throwing space	92.9%	7.1%	
		Percentage within attacking type	64.6%	23.7%	57.5%
		Total percentage	53.4%	4.1%	
		Standardized residuals	2.3	−5.1	
		Number of actions	624	130	754
		Total percentage	82.6%	17.4%	

χ² (2) = 77.49, Cramér’s V = 0.32; *p* < 0.001.

**Table 2:** Relation between the attacking type (direct actions or counter-attacks) and lateral structure of fighting (symmetrical [R-R and L-L] and asymmetrical [R-L and L-R]) (*n* = 755).

				Attacking Type		
				Direct Actions	Counter-Attacks	Total
Lateral structure of fighting	Symmetrical	R-R	Number of actions	146	6	
			Percentage within lateral structure of fighting	96.1%	3.9%	
			Percentage within attacking type	23.4%	4.6%	12.7%
			Total percentage	19.3%	0.8%	
			Standardized residuals	1.8	−4.0	
		L-L	Number of actions	86	10	
			Percentage within lateral structure of fighting	89.6%	10.4%	33.8%
			Percentage within attacking type	13.8%	7.6%	
			Total percentage	11.4%	1.3%	
			Standardized residuals	−0.7	−1.6	
			Number of actions	232	16	
			Percentage within lateral structure of fighting	93.5%	6.5%	
			Percentage within attacking type	37.2%	12.2%	32.8%
			Total percentage	30.7%	2.1%	
			Standardized residuals	1.9	−4.1	
	Asymmetrical	R-L	Number of actions	186	66	
			Percentage within lateral structure of fighting	73.8%	26.2%	
			Percentage within attacking type	29.8%	50.4%	33.4%
			Total percentage	24.6%	8.7%	
			Standardized residuals	−1.5	3.4	
		L-R	Number of actions	206	49	
			Percentage within lateral structure of fighting	80.8%	19.2%	
			Percentage within attacking type	33.0%	37.4%	20.1%
			Total percentage	27.3%	6.5%	
			Standardized residuals	−0.3	0.7	
			Number of actions	392	115	
			Percentage within lateral structure of fighting	77.3%	22.7%	
			Percentage within attacking type	62.8%	87.8%	67.2%
			Total percentage	51.9%	15.2%	
			Standardized residuals	−1.3	2.9	
	Number of actions			624	131	755
	Total percentage			82.6%	17.4%	

χ² (1) = 30.59, Cramér’s V = 0.20; *p* < 0.001.

**Table 3:** Relation between the throwing space (forward throw area and backward thrown area) and the lateral structure of fighting (symmetrical [R-R and L-L] and asymmetrical [R-L and L-R]) (*n* = 754).

				Throwing Space		
				Forward Throw Area	Backward Thrown Area	Total
Lateral structure of fighting	Symmetrical	R-R	Number of actions	103	49	
			Percentage within lateral structure of fighting	67.8%	32.2%	
			Percentage within throwing space	23.7%	15.3%	20.2%
			Total percentage	13.7%	6.5%	
			Standardized residuals	1.7	−1.9	
		L-L	Number of actions	58	38	
			Percentage within lateral structure of fighting	60.4%	39.6%	12.7%
			Percentage within throwing space	13.4%	11.9%	
			Total percentage	7.7%	5.0%	
			Standardized residuals	0.4	−0.4	
			Number of actions	161	87	
			Percentage within lateral structure of fighting	64.9	35.1%	
			Percentage within throwing space	37.1%	27.2%	32.9%
			Total percentage	21.4%	11.5%	
			Standardized residuals	1.5	−1.8	
	Asymmetrical	R-L	Number of actions	134	117	
			Percentage within lateral structure of fighting	53.4%	46.6%	
			Percentage within throwing space	30.9%	36.6%	33.3%
			Total percentage	17.8%	15.5%	
			Standardized residuals	−0.9	1	
		L-R	Number of actions	139	116	
			Percentage within lateral structure of fighting	54.5%	45.5%	
			Percentage within throwing space	32.0%	36.3%	33.8%
			Total percentage	18.4%	15.4%	
			Standardized residuals	−0.6	0.7	
			Number of actions	273	233	
			Percentage within lateral structure of fighting	54.0%	46.0%	
			Percentage within throwing space	62.9%	72.8%	67.1%
			Total percentage	36.2%	30.9%	
			Standardized residuals	−1.1	1.2	
	Number of actions			434	320	
	Total percentage			57.6%	42.4%	

χ² (2) = 8.2, Cramér’s V = 0.1; *p* = 0.04.

**Table 4:** Relation between the lateral structure of fighting (symmetrical [R-R and L-L] and asymmetrical [R-L and L-R]) and the category of the technique (*n* = 755).

				Category of the Technique										
				Turn_F2	Turn_F1	WT_IpExB1	WT_IpInB1	WT_ClaInB1	WT_ClaExB1	WT_ClaExF1	WT_2	WT_1	SP_FwBack	Total
Lateral structure of fighting	Symmetrical	R-R	Number of actions	61	19	31	9	5	2	9	7	1	8	152
			Percentage within lateral structure of fighting	40.1%	12.5%	20.4%	5.9%	3.3%	1.3%	5.9%	4.6%	0.7%	5.3%	
			Percentage within the category of the technique	31.3%	14.1%	55.4%	26.5%	7.7%	1.6%	42.9%	8.3%	14.3%	24.2%	20.1%
			Total percentage	8.1%	2.5%	4.1%	1.2%	0.7%	0.3%	1.2%	0.9%	0.1%	1.1%	
			Standardized residuals	3.5	−1.6	5.9	0.8	−2.2	−4.6	2.3	−2.4	−0.3	0.5	
		L-L	Number of actions	33	9	18	13	2	1	8	4	1	7	96
			Percentage within lateral structure of fighting	34.4%	9.4%	18.8%	13.5%	2.1%	1.0%	8.3%	4.2%	1.0%	7.3%	
			Percentage within the category of the technique	16.9%	6.7%	32.1%	38.2%	3.1%	0.8%	38.1%	4.8%	14.3%	21.2%	12.7%
			Total percentage	4.4%	1.2%	2.4%	1.7%	0.3%	0.1%	1.1%	0.5%	0.1%	0.9%	
			Standardized residuals	1.6	−2.0	4.1	4.2	−2.2	−3.7	3.3	−2.0	0.1	1.4	
			Number of actions	94	28	49	22	7	3	17	11	2	15	248
			Percentage within lateral structure of fighting	37.9%	11.3%	19.8%	8.9%	2.8%	1.2%	6.9%	4.4%	2.3	6.0%	
			Percentage within the category of the technique	48.2%	20.7%	87.5%	64.7%	10.8%	2.4%	81.0%	13.1%	0.8%	45.5%	32.8%
			Total percentage	12.5%	3.7%	6.5%	2.9%	0.9%	0.4%	2.3%	1.5%	28.6%	2.0%	
			Standardized residuals	3.7	−2.5	7.1	3.2	−3.1	−5.9	3.8	−3.2	−0.2	1.3	
	Asymmetrical	R-L	Number of actions	43	56	5	5	25	67	1	37	4	9	252
			Percentage within lateral structure of fighting	17.1%	22.2%	2.0%	2.0%	9.9%	26.6%	0.4%	14.7%	1.6%	3.6%	
			Percentage within the category of the technique	22.1%	41.5%	8.9%	14.7%	38.5%	53.6%	4.8%	44.0%	57.1%	27.3%	33.4%
			Total percentage	5.7%	7.4%	0.7%	0.7%	3.3%	8.9%	0.1%	4.9%	.5%	1.2%	
			Standardized residuals	−2.7	1.6	−3.2	−1.9	0.7	3.9	−2.3	1.7	1.1	−0.6	
		L-R	Number of actions	58	51	2	7	33	55	3	36	1	9	255
			Percentage within lateral structure of fighting	22.7%	20.0%	0.8%	2.7%	12.9%	21.6%	1.2%	14.1%	0.4%	3.5%	
			Percentage within the category of the technique	29.7%	37.8%	3.6%	20.6%	50.8%	44.0%	14.3%	42.9%	14.3%	27.3%	33.8%
			Total percentage	7.7%	6.8%	0.3%	0.9%	4.4%	7.3%	0.4%	4.8%	0.1%	1.2%	
			Standardized residuals	−1.0	0.8	−3.9	−1.3	2.4	2.0	−1.5	1.4	−0.9	−0.6	
			Number of actions	101	107	7	12	58	122	4	73	5	18	507
			Percentage within lateral structure of fighting	19.9%	21.1%	1.4%	2.4%	11.4%	24.1%	0.8%	14.4%	1.0%	3.6%	
			Percentage within the category of the technique	51.8%	79.3%	12.5%	35.3%	89.2%	97.6%	19.0%	86.9%	71.4%	54.5%	67.2%
			Total percentage	13.4%	14.2%	0.9%	1.6%	7.7%	16.2%	0.5%	9.7%	0.7%	2.4%	
			Standardized residuals	−2.6	1.7	−5.0	−2.3	2.2	4.2	−2.7	2.2	0.1	−0.9	
	Number of actions			195	135	56	34	65	125	21	84	7	33	
	Total percentage (%)			25.8%	17.9%	7.4%	4.5%	8.6%	16.6%	2.8%	11.1%	0.9%	4.4%	

χ² (9) = 227.5, Cramér’s V = 0.54; *p* < 0.001. Turn_F2: Techniques of turning, forward throw, and two supporting legs. Turn_F1: Techniques of turning, forward throw, and one supporting leg. WT_IpExB1: Techniques without turning, ipsilateral leg direction, external zone, backward throw, and one supporting leg. WT_IpInB1: Techniques without turning, ipsilateral leg direction, inner zone, backward throw, and one supporting leg. WT_ClaInB1: Techniques without turning, contralateral leg direction, inner zone, backward throw, and one supporting leg. WT_ClaExB1: Techniques without turning, contralateral leg direction, external zone, backward throw, and one supporting leg. WT_ClaExF1: Techniques without turning, contralateral leg direction, external zone, forward throw, and one supporting leg. WT_2: Techniques without turning, forward or backward throw, and two supporting legs. WT_1: Techniques without turning, forward or backward throw, and one supporting leg. SP_FwBack: Techniques of supine position, forward throw, and back support. Note: Considering just symmetrical and asymmetrical categories, two boxes (10%) had an expected frequency lower than five. While considering the four subgroups, eight boxes (20%) had an expected frequency lower than five.

## References

[1] Grouios G. (2004). Motoric dominance and sporting excellence: training versus heredity. Percept. Mot. Skills.

[2] Miarka B., Cury R., Julianetti R., Battazza R., Julio U.F., Calmet M., Franchini E. (2014). A comparison of time-motion and technical-tactical variables between age groups of female judo matches. J. Sports Sci..

[3] Miarka B., Branco B.H.M., Del Vecchio F.B., Camey S., Franchini E. (2015). Development and validation of a time-motion judo combat model based on the Markovian Processes. Int. J. Perform. Anal. Sport.

[4] Courel J., Franchini E., Femia P., Stankovic N., Education P. (2014). Effects of kumi-kata grip laterality and throwing side on attack effectiveness and combat result in elite judo athletes. Int. J. Perform. Anal. Sport.

[5] Boguszewski D. (2011). Relationships between the rules and the way of struggle applied by top world male judoists. Arch. Budo.

[6] Adam M., Smaruj M. (2013). The indices of technical-tactical preparation of the World’s Judo Champions in Tokyo 2010 as an assessment criterion for individual training. Arch. Budo.

[7] Calmet M., Miarka B., Franchini E. (2010). Modeling of grasps in judo contests. Int. J. Perform. Anal. Sport.

[8] Franchini E., Sterkowicz S., Meira C.M.J., Gomes F.R.F., Tani G. (2008). Technical variation in a sample of high level judo players. Percept. Mot. Skills.

[9] Calmet M., Trezel N., Ahmaidi S. (2006). Survey of system of attacks by judoka in regional and interregional matches. Percept. Mot. Skills.

[10] Calmet M., Ahmaidi S. (2004). Survey of advantages obtained by judoka in competition by level of practice. Percept. Mot. Skills.

[11] Abdel-Raouf Y.Y., Abdelhalem A.M. (2011). Skillful and tactical analysis of the World Judo Senior Championship—Japan 2010 According to the new amendments of the regulations. World J. Sport Sci..

[12] Adam M., Radosław L., Smaruj M. (2012). Directions and ways of executing judo throws during judo contests as a control criterion of an individual’s training versatility. Balt. J. Heal. Phys. Act..

[13] Miarka B., Hayashida C.R., Julio U.F., Calmet M., Franchini E. (2011). Objectivity of FRAMI-Software for Judo Match Analysis. Int. J. Perform. Anal. Sport.

[14] Marcon G., Franchini E., Jardim J.R., Barros Neto T.L. (2010). Structural Analysis of Action and Time in Sports: Judo. J. Quant. Anal. Sport.

[15] Iglesias-Soler E., Mayo X., Dopico X., Fernández-Del-Olmo M., Carballeira E., Fariñas J., Fernández-Uribe S. (2018). Effects of bilateral and non-dominant practices on the lateral preference in judo matches. J. Sports Sci..

[16] Dopico X., Iglesias-Soler E., Carballeira E. (2014). Classification of judo motor skills: Tactical and motor criteria approach. Arch. Budo.

[17] Sterkowicz S., Sacripanti A., Przybycien K.S., Sterkowicz-Przybycień K. (2013). Techniques frequently used during London Olympic judo tournaments: A biomechanical approach. Arch. Budo.

[18] Raymond M., Pontier D., Dufour A.B., Møller A.P. (1996). Frequency-dependent maintenance of left handedness in humans. Proc. Biol. Sci..

[19] Mikheev M., Mohr C., Afanasiev S., Landis T., Thut G. (2002). Motor control and cerebral hemispheric specialization in highly qualified judo wrestlers. Neuropsychologia.

[20] Dopico X., Iglesias-Soler E., Carballeira E., Mayo X., Ardá A., González-Freire M. (2014). The relationship between motoric dominance and functional dominance while executing judo techniques: A study on laterality. Arch. Budo.

[21] Adam M., Tyszkowski S., Smaruj M. (2011). The Contest Effectiveness of the Men’s National Judo Team of Japan, and Character of Their Technical-Tactical Preparation during the World Judo Championships 2010. Balt. J. Heal. Phys. Act..

[22] Kajmovic H., Radjo I. (2014). A Comparison of Gripping Configuration and Throwing Techniques Efficiency Index in Judo Between Male and Female Judoka During Bosnia and Herzegovina Senior State Championships. Int. J. Perform. Anal. Sport.

[23] Miarka B., Fukuda H.D., Del Vecchio F.B., Franchini E. (2016). Discriminant analysis of technical-tactical actions in high-level judo athletes. Int. J. Perform. Anal. Sport.

